# Rational designed Fe-ZIFs@CoP nanoplatforms for photothermal-enhanced ROS-mediated tumor therapy

**DOI:** 10.3389/fbioe.2024.1361347

**Published:** 2024-01-31

**Authors:** Chen Wang, Shufang Ning, Jinling Mai, Shanyu Zhao, Wenwei Jiang, Junjie Pan, Feifei Wu, Qiuju Liu, Qinle Zhang

**Affiliations:** ^1^ Maternal and Child Health Hospital of Guangxi Zhuang Autonomous Region, Nanning, China; ^2^ Guangxi Medical University Cancer Hospital, Nanning, China; ^3^ The First Affiliated Hospital of Guangxi Medical University, Nanning, China

**Keywords:** Fe-based MOFs, ROS, CoP, Fenton reaction, photothermal effect

## Abstract

Metal-organic frameworks (MOFs), with biocompatible and bio-friendly properties, exhibit intriguing potential for the drug delivery system and imaging-guided synergistic cancer theranostics. Even though tremendous attention has been attracted on MOFs-based therapeutics, which play a crucial role in therapeutic drugs, gene, and biomedical agents delivery of cancer therapy, they are often explored as simple nanocarriers without further “intelligent” functions. Herein, Fe-doped MOFs with CoP nanoparticles loading were rationally designed and synthesized for photothermal enhanced reactive oxygen species (ROS)-mediated treatment. Fe-ZIFs@CoP could generate efficient ROS through the Fenton reaction while depleting glutathione for amplifying oxidative stress. Particularly, due to the photothermal effect of Fe-ZIFs@CoP, the hyperthermia generated by as-synthesized Fe-ZIFs@CoP facilitated the advanced performance of the Fenton effect for a high amount of ROS generation. The promising “all-in-one” synergistic MOFs platform herein reported provides some prospects for future directions in this area.

## 1 Introduction

Tumors are harmful to human health featuring with increased mortality and causing millions of deaths worldwide (Zhang et al., 2023a; Zhao et al., 2023a; Zhang et al., 2023b; Gil et al., 2023; Yong et al., 2023). Compared with early-stage tumors, malignant tumors are not easy to clear, but easy to relapse and metastasize, destroying the structure and function of normal tissues and organs, causing complications such as necrosis, bleeding, and co-infection, and eventually leading to organ failure and death (Chang et al., 2022; Shan et al., 2022; Diao and Liu, 2023; Li et al., 2023; Wang et al., 2023).Due to the advances in nanotechnology, rationally designed nanomaterials provide a promising approach for tumor therapy (Wang et al., 2021a; Xujiang et al., 2021; Yu et al., 2021; Zhang et al., 2021; Jiang et al., 2023). Significantly, metal-organic frameworks (MOFs) with multi-functional biological properties, including cellular uptake, cytotoxicity, biodistribution, as well as blood circulation time are widely investigated in drug delivery systems (DDS) and other biomedical applications (Cai et al., 2019; Gao et al., 2019; Gong et al., 2019; Hao et al., 2019; Yang et al., 2023).

MOFs are a battery of crystalline porous materials, composed of metal-containing cores and organic linkers (Wang et al., 2019; Bao et al., 2021; Liang et al., 2021; Mengyun et al., 2021). Due to the compositional and structural adjustability, MOFs with some appealing characteristics including versatile framework structure, high surface area, tunable crystal size, large internal pore volumes, rich surface chemistries, and excellent biocompatibility have been explored in the diverse range, such as gas storage and separation, catalysis, energy conversion, sensors, semiconductivity, and biomedicine (Wang et al., 2021b; Weiwei et al., 2021; Xiaogang et al., 2021; Bian et al., 2022; Chen et al., 2022; Chung et al., 2022; Guo et al., 2022). To date, utilizing the collective properties and advanced performance of MOFs is promising. Liang *et al.* reported a defect-rich Ti-based MOF (D-MOF(Ti)) with greatly improved sonosensitizing effect for enhanced sonodynamic therapy. The as-synthesized D-MOF(Ti) exhibited a superior reactive oxygen species (ROS) yield under ultrasound irradiation due to its narrow bandgap, which principally improves the ultrasound-triggered electron-hole separation. ROS are molecules that contain and participate in electron transfer, which play an important role in maintaining tissue homeostasis, regulating signal transduction, and promoting cell injury and death. In particular, hydroxyl radical (•OH) is one of the most active oxygen species with high reaction activity and strong oxidation ability. However, the overexpressed glutathione (GSH) with antioxidation impaired the ROS effects. It is important to deplete GSH to amplify the oxidation effect. MOFs, on the one hand, can serve as nanocarriers of contrast agents due to their oriented porous structure. On the other hand, MOFs themselves can be functionalized as therapeutics due to their intrinsic properties (Li et al., 2022a; Li et al., 2022b; Jadhav et al., 2022; Jiang et al., 2022; Wu et al., 2022; Fu et al., 2023; Jia et al., 2023). In addition, MOFs are tumor microenvironment-responsive nanoplatforms due to their potential biodegradability (Yu et al., 2023a; Yu et al., 2023b; Zhao et al., 2023b; Fang et al., 2023; Xu et al., 2023). Among these, the unique features of Fe-doping ZIFs with functional nanoparticles loading with advanced Fenton reaction properties and GSH depletion activities are promising in tumor therapy.

Traditional cancer therapies include chemotherapy, radiotherapy, surgical resection, and molecularly targeted therapy, in which chemotherapy and radiotherapy are still the main methods in clinics. However, chemotherapy and radiotherapy present obvious limitations, such as whole-body adverse reactions and significant toxic side effects from chemotherapy, inevitable micro-tumor remnants and damage to normal tissues around tumors by ionizing radiation, a high recurrence rate of surgical resection, and undesirable therapeutic efficacy of molecularly targeted therapy. Thus, some other drug-free and non-surgical treatments, such as photo therapy, chemodynamic therapy, sonodynamic therapy, and immunotherapy are rapidly developed for higher anticancer efficacy (Zhou et al., 2023a; Zhu et al., 2023a; Bai et al., 2023; Zhou et al., 2023b; Zhu et al., 2023b; Zhao et al., 2023c; Ding et al., 2023).

Herein, Fe-doped ZIFs with CoP nanoparticles loaded nanoplatforms were rationally designed and synthesized for photothermal enhanced ROS-mediated treatment. Fe-ZIFs@CoP could generate efficient ROS through the Fenton reaction while depleting glutathione for amplifying oxidative stress. Particularly, due to the photothermal effect of Fe-ZIFs@CoP, the hyperthermia generated by as-synthesized Fe-ZIFs@CoP facilitated the advanced performance of the Fenton effect for a high amount of ROS generation. The promising “all-in-one” synergistic Fe-ZIFs@CoP nanoplatforms herein reported provide some prospects for future directions in this area.

## 2 Experimental section

### 2.1 Materials

Zinc nitrate hexahydrate (Zn(NO_3_)_2_·6H_2_O), cobalt acetylacetonate methyl (Co(acac)_3_), iron nitrate nonahydrate (Fe(NO_3_)_3_·9H_2_O), 5,5-dithiobis-(2-nitrobenzoic acid) (DTNB), 3,3′,5,5′-tetramethyl-benzidine (TMB), alcohol, methyl imidazole, ethanol, hexachloride, tri-n-octylphosphine, cyclohexane, ethyl alcohol, 4,4′-Sulfonyldiphenol, triethylamine, and polyethylene glycol (PEG) were purchased from Sigma-Aldrich. Calcein acetoxymethyl ester (Calcein AM), 2′,7′-dichlorofluorescein diacetate (DCFH-DA), Hematoxylin and Eosin Staining Kit (H&E), methyl thiazolyl-diphenyl-tetrazolium bromide (MTT), and propidium iodide (PI) were obtained from Beyotime Inst. Biotech.

### 2.2 Characterization

The X-ray diffraction (XRD) patterns were conducted using a Rigaku D/max-TTR-III diffractometer (Cu-Ka radiation, λ = 0.154 nm). Transmission electron microscopy (TEM) was utilized for observing the morphology of as-synthesized materials with an FEI Tecnai G2 S-Twin transmission electron microscope. ESCALAB 250 instrument was presented for investigating X-ray photoelectron spectroscopy (XPS) spectra. Inductively coupled plasma-optical emission spectrometry (ICP-OES) was conducted for element quantitative analysis (Agilent 725).

### 2.3 Synthesis of Fe-ZIFs@CoP

At first, 458 mg of Zn(NO_3_)_2_·6H_2_O and 124 mg of Fe(NO_3_)_3_·9H_2_O were dissolved in 30 mL methanol to form a uniform solution, and then 1,314 mg of 2-methylimidazole was dissolved in 30 mL methanol. The two solutions were mixed and stirred at 35 ^o^C for 4 h. The samples were washed with methanol, dried, and obtained Fe-ZIFs. Then, 25 mg Fe-ZIFs were added in 11 mL methanol for dispersion. And 27 mg of hexachloride and 61 mg of 4,4′-sulfonyl diphenol were dispersed in 9 mL methanol. The above solution was dropwise added and stirred evenly. Then 30 μL of triethylamine was added and stirred for 18 h. The product was centrifuged and dried under a vacuum (Fe-ZIFs@PZS). The obtained powder was heated to 400°C in a flowing Ar atmosphere for 3 h (Sintered Fe-ZIFs).

Next, 128 mg of cobalt acetylacetone was dissolved in 10 mL oleamine, magnetically stirred, heated to 120 °C under N_2_ environment, and maintained for 1 h. Then, 1 mL of tri-n-octylphosphine was added. The mixture was quickly heated to 280°C and held for 2 h. The products were collected by centrifugation with ethanol and cyclohexane and dried in a vacuum (CoP nanoparticles). Finally, 10 mg of the sintered Fe-ZIFs was dispersed in 10 mL methanol and 10 mg of CoP was dispersed in 10 mL cyclohexane, mixing and stirring for 4 h, and then centrifuged and dried in a vacuum, obtaining Fe-ZIFs@CoP.

### 2.4 Fenton reaction properties

To analyze the Fenton reaction properties, a mixture solution was prepared in 3 mL PBS (pH 5.5) with TMB as substrate and Fe-ZIFs@CoP (100 μg mL^−1^) as catalyst under H_2_O_2_ condition. The Fenton effect properties of Fe-ZIFs@CoP were investigated by using different concentrations of H_2_O_2_ as substrate, monitoring the specific absorbance around 650 nm after different reaction times. Meanwhile, the absorption spectra of oxTMB in Fe-ZIFs@CoP solution (100 μg mL^−1^) were obtained by fixing the reaction time at 5 min under control, different pH, and different temperatures.

### 2.5 GSH consumption properties

A mixture of Fe-ZIFs@CoP (100 μg mL^−1^), GSH (10 mM), and PBS containing DTNB was used as the solution to be tested. After different times, the absorbance at 412 nm was recorded by UV-vis spectrophotometer.

### 2.6 Photothermal properties

Fe-ZIFs@CoP dispersions with different concentrations were irradiated with an 808 nm laser for 400 s. The temperature variation over time was photographed with an infrared thermal camera. The changed irradiation time and laser power condition were also recorded. To characterize the photothermal stability, Fe-ZIFs@CoP dispersion was irradiated with laser power of 0.8 W cm^−2^ for 400 s, then cooled to room temperature. The heating and cooling processes were recorded for three cycles. The temperature variation in the above heating and cooling process was recorded to calculate the photothermal conversion efficiency (η):

$$\eta =\frac{hA\left(T\max\;―T\text{surr}\right)―\text{Qs}}{I\left(1―10^{―\;A_{\lambda }}\;\right)}$$

*h* is the thermal conversion efficiency, *S* is the surface area of the test vessel, *T*_max_ is the temperature of the sample solution when it reaches thermal equilibrium, *I* is the power density of the laser, and *A*_λ_ is the absorbance of the sample solution at 808 nm.

### 2.7 *In vitro* experiments

The CT26 colorectal cancer cell line and L929 fibroblast cell line were obtained from FDCC (Ruilu in Shanghai, China). A standard MTT assay was used to investigate the therapeutic effect and safety of Fe-ZIFs@CoP. CT26 cells were inoculated in 96-well plates overnight. Then, the cells were divided into four treatment groups: (1) control, (2) NIR (0.8 W cm^−2^, 5 min), (3) Fe-ZIFs@CoP (200 μg mL^−1^), and (4) Fe-ZIFs@CoP + NIR (0.8 W cm^−2^, 5 min). In addition, L929 cells were incubated similarly to the above processes. Differently, only Fe-ZIFs@CoP were added, followed by incubation for 12 h and 24 h, and then MTT was added to evaluate the safety of materials. For evaluation of the therapeutic effects of Fe-ZIFs@CoP, the cells were divided into the following treatment groups: (1) control, (2) NIR (0.8 W cm^−2^, 5 min), (3) Fe-ZIFs@CoP (200 μg mL^−1^), and (4) Fe-ZIFs@CoP + NIR (0.8 W cm^−2^, 5 min). After that, all the cells were washed 3 times with PBS. Subsequently, 1 mL of DAPI was added to each well to stain the nucleus. To assess the level of ROS produced by Fe-ZIFs@CoP in the cell, after the above treatments, 1 mL of DCFH-DA (5 μM) was added to each well. The treated cells were photographed with CLSM. To identify living and dead cells, the CT26 cells were incubated and treated as described above. Then, 1 mL of calcein-AM (5 µM) was added to each well of a 6-well plate. After incubation for 15 min, the cells were washed, and 1 mL of PI (5 µM) was added to co-incubate with them for another 15 min. Followed by rinsing and fixing with glutaraldehyde (2.5%), the staining images of cells were taken by CLSM.

### 2.8 *In vivo* experiments

The animal study protocol was approved by the Ethics Committee of Guangxi Medical University Cancer Hospital (protocol code KY 2022–129/130 and approved on 25 February 2022) for studies involving animals. When the tumor volume reached 100 mm^3^, 200 µL of Fe-ZIFs@CoP dispersion was injected into mice *via* the tail vein. For evaluating the *in vivo* assessment of the therapeutic effect, twenty mice with a tumor size of 100 mm^3^ were divided into the following treatment groups: (1) control, (2) NIR, (3) Fe-ZIFs@CoP, and (4) Fe-ZIFs@CoP + NIR. Typically, mice in groups (3) and (4) were injected with 0.2 mL of Fe-ZIFs@CoP solution (2 mg kg^−1^) *via* the tail vein. Then, mice in groups (3) and (4) were irradiated by 808 nm (0.8 W cm^−2^) light at certain time points for therapy. Thereafter, the volume and weight of tumors in different groups were measured every 2 days to assess the treatment effect. After 14 days of treatment, major organs and tumors were dissected and stained with H&E for histological examination.

### 2.9 Statistical analysis

Quantitative data were conducted as mean ± standard deviation (mean ± S.D.), and the Student’s t*-*test was used to analyze all experimental data, where the statistical significance *p < 0.05 represents significant, **p < 0.01 represents moderately significant, and ***p < 0.001 represents highly significant.

## 3 Result and discussion

### 3.1 Material characterization

**FIGURE 1:**
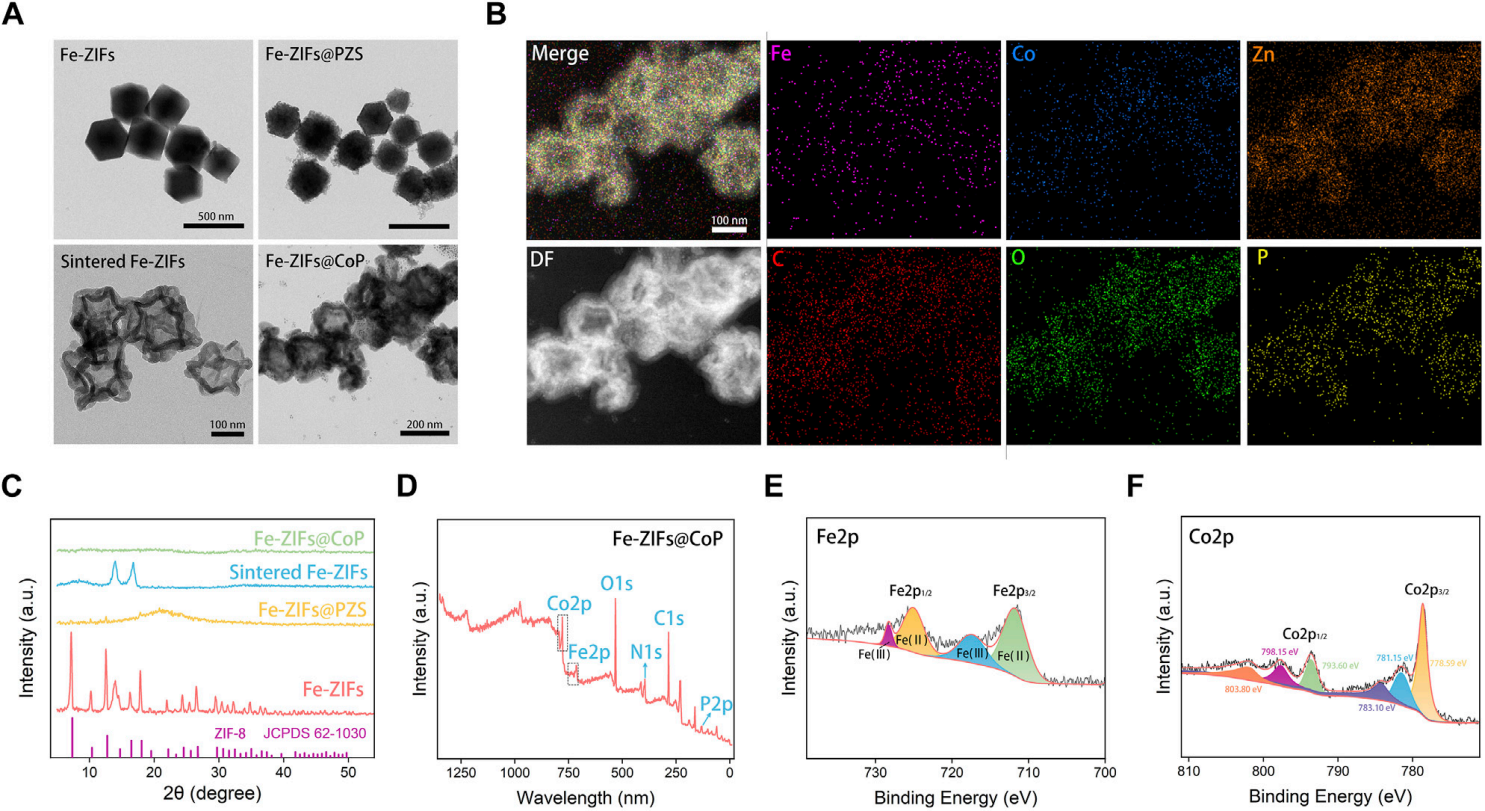
**(A)** TEM images of Fe-ZIFs, Fe-ZIFs@PZS, sintered Fe-ZIFs, and Fe-ZIFs@CoP. **(B)** Element mapping of ZIFs@CoP. **(C)** XRD patterns of Fe-ZIFs, Fe-ZIFs@PZS, sintered Fe-ZIFs, and Fe-ZIFs@CoP. **(D–F)** XPS spectrum of Fe-ZIFs@CoP.

The basic characterization of Fe-ZIFs@CoP samples is summarized in Figure 1. The TEM image in Figure 1A presents the morphology of Fe-ZIFs, Fe-ZIFs@PZS, sintered Fe-ZIFs, and Fe-ZIFs@CoP. As shown, both Fe-ZIFs and Fe-ZIFs@PZS nanoparticles were dodecahedrons with average diameters of approximately 325 nm. After sintering, the sintered Fe-ZIFs exhibited average diameters of around 160 nm. Finally, the CoP nanoparticles were loaded. The size distribution of Fe- ZIFs@CoP was exhibited in Supplementary Figure S1. Also, the step-wise zeta potentials of Fe-ZIFs, Fe-ZIFs@PZS, sintered Fe-ZIFs, and Fe-ZIFs@CoP was shown in Supplementary Figure S2. The changed zeta potentials step-by-step demonstrated the successful synthesis of Fe-ZIFs@CoP. The composition of Fe-ZIFs@CoP was clearly illustrated in the high-angle annular dark field-scanning TEM image and corresponding elemental mappings, which indicates the presence of Fe, Zn, Co, C, O, and P, proving that Fe were distributed uniformly, and the hollow structure is visible (Figure 1B).

The XRD patterns of Fe-ZIFs, Fe-ZIFs@PZS, sintered Fe-ZIFs, and Fe-ZIFs@CoP were shown in Figure 1C, respectively. The diffraction peaks of Fe-ZIFs in the XRD patterns corresponded with the standard cards of the ZIF structure, thus confirming the existence of the ZIF structure (JCPDS No. 62–1,030). Owing to the sintering and loading of CoP, the XRD patterns of others did not exhibit significant ZIFs structure. The surface composition and chemical state of Fe-ZIFs@CoP were analyzed by using XPS in detail. In the survey XPS spectrum, the elemental composition of Fe and Co was demonstrated (Figure 1D). The high-resolution XPS spectrum of Fe 2p showed peaks at 728.6, 725.3, 718.2, and 710.8 eV, which can be assigned to Fe 2p_1/2_ and Fe 2p_3/2_, respectively (Figure 1E). XPS spectrum of Co 2p (Figure 1F), the primary peaks at 803.80, 798.15, 793.60, 783.10, 781.15, and 728.59 eV were assigned to Co 2p_1/2_ and Co 2p_3/2_, respectively. The varied valence of Fe and Co ions determined the high Fenton reaction properties. Finally, we also investigated the stability properties of Fe-ZIFs@CoP within 8 days (Supplementary Figure S3), which showed well stability properties for further application.

### 3.2 Fenton reaction properties

**FIGURE 2:**
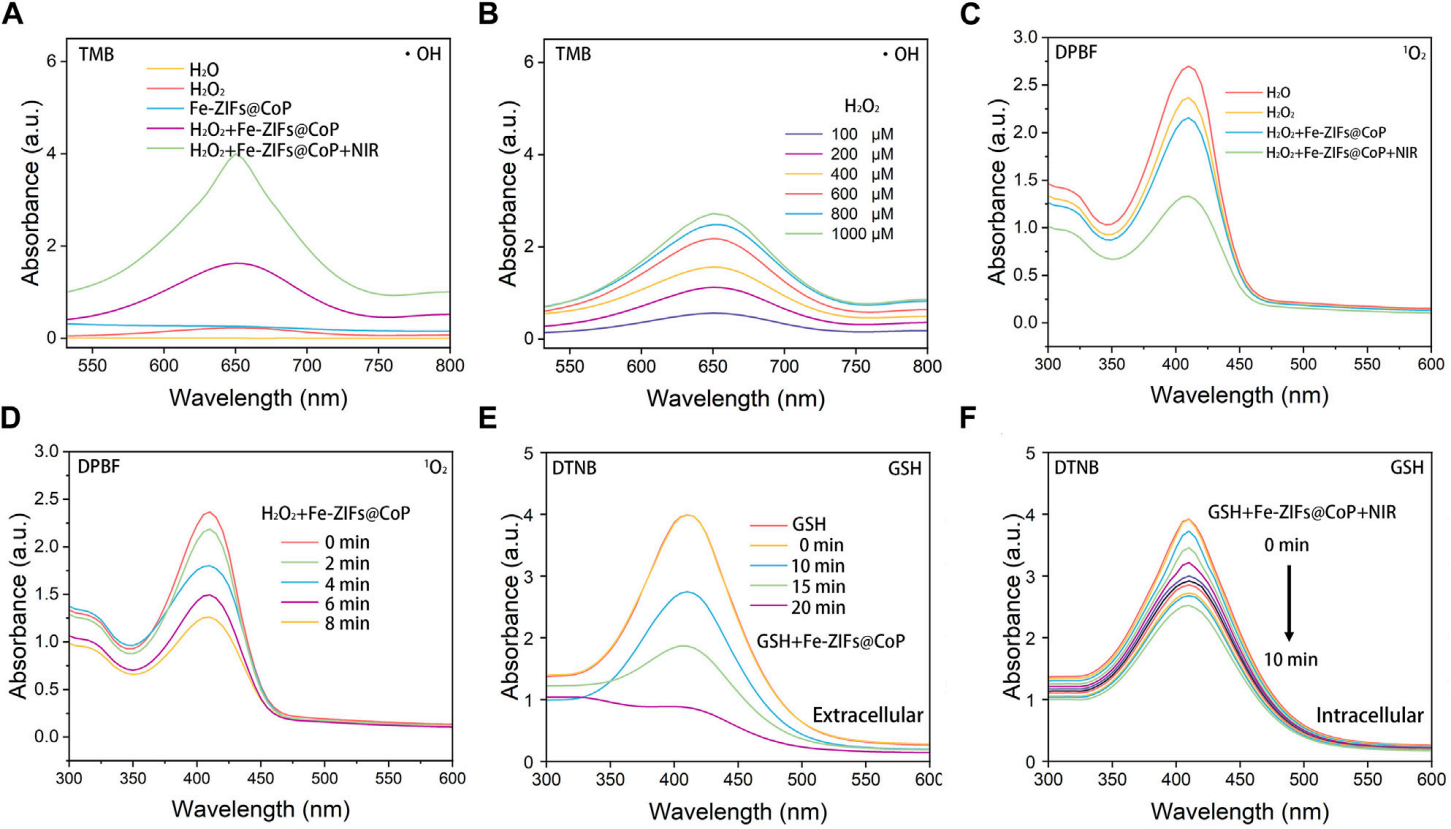
**(A)** UV-vis absorption spectra of TMB catalyzed by different groups. **(B)** UV-vis absorption spectra of TMB catalyzed by Fe-ZIFs@CoP under different concentrations. **(C)** The generation of ^1^O_2_ in different groups. **(D)** The generation of ^1^O_2_ by Fe-ZIFs@CoP under different concentrations. **(E)** UV-vis absorption spectra of GSH consumption by Fe-ZIFs@CoP with time increase. **(F)** UV-vis absorption spectra of GSH consumption by Fe-ZIFs@CoP + NIR with time increase.

The Fe-ZIFs@CoP nanoplatforms possess both Fenton reaction properties and GSH depletion activities, which could catalyze H_2_O_2_ to ∙OH and generate ^1^O_2_ for ROS-mediated therapy, and deplete GSH for amplifying oxidative stress Additionally, the photothermal effect of Fe-ZIFs@CoP can enhance both ROS generation ability. To gain more insights into the Fenton reaction properties of Fe-ZIFs@CoP, TMB, and H_2_O_2_ were used as substrates. Figure 2A showed the change in the absorbance of oxidated TMB (oxTMB, at 650 nm) in different groups. There was no relevant peak detected in the H_2_O, H_2_O_2_, and Fe-ZIFs@CoP groups, while H_2_O_2_+Fe-ZIFs@CoP and H_2_O_2_+Fe-ZIFs@CoP + NIR groups showed well ability of •OH generation. Furthermore, upon NIR irradiation, a higher •OH yield was obtained due to the photothermal effect. In addition, upon increased concentrations of Fe-ZIFs@CoP, the concentration-related production of •OH was obtained from the gradually raised signal strength (Figure 2B). Furthermore, as another catalytic product, the ^1^O_2_ can be detected by DPBF, which exhibited a specific peak at 420 nm (Figure 2C). Compared with the H_2_O, H_2_O_2_, and Fe-ZIFs@CoP groups, H_2_O_2_+Fe-ZIFs@CoP and H_2_O_2_+Fe-ZIFs@CoP + NIR groups showed enhanced ^1^O_2_ generation ability. There is a significant peak decrease of the H_2_O_2_+Fe-ZIFs@CoP + NIR group, which exhibited a huge contrast with the Fe-ZIFs@CoP group, confirming the promotion effect of NIR irradiation. With the increased time, the absorbance peak decreased as time passed, indicating the gradual generation of ^1^O_2_ (Figure 2D).

The overexpressed antioxidant GSH will react with the generated ROS, thus impairing the ROS-mediated therapeutic effect. Hence, GSH depletion is important for the high efficiency of ROS-mediated anti-tumor therapy. The GSH depletion ability of Fe-ZIFs@CoP was investigated using the GSH indicator Figure 2E. In the presence of Fe-ZIFs@CoP, the GSH content is gradually decreased with increased time. Under 808 nm laser irradiation, the GSH depletion performance enhanced with time increased due to the photothermal effect. The decrease of GSH by Fe-ZIFs@CoP is favorable for maintaining the high level of ROS in the tumor microenvironment.

### 3.3 Photothermal effect properties

**FIGURE 3:**
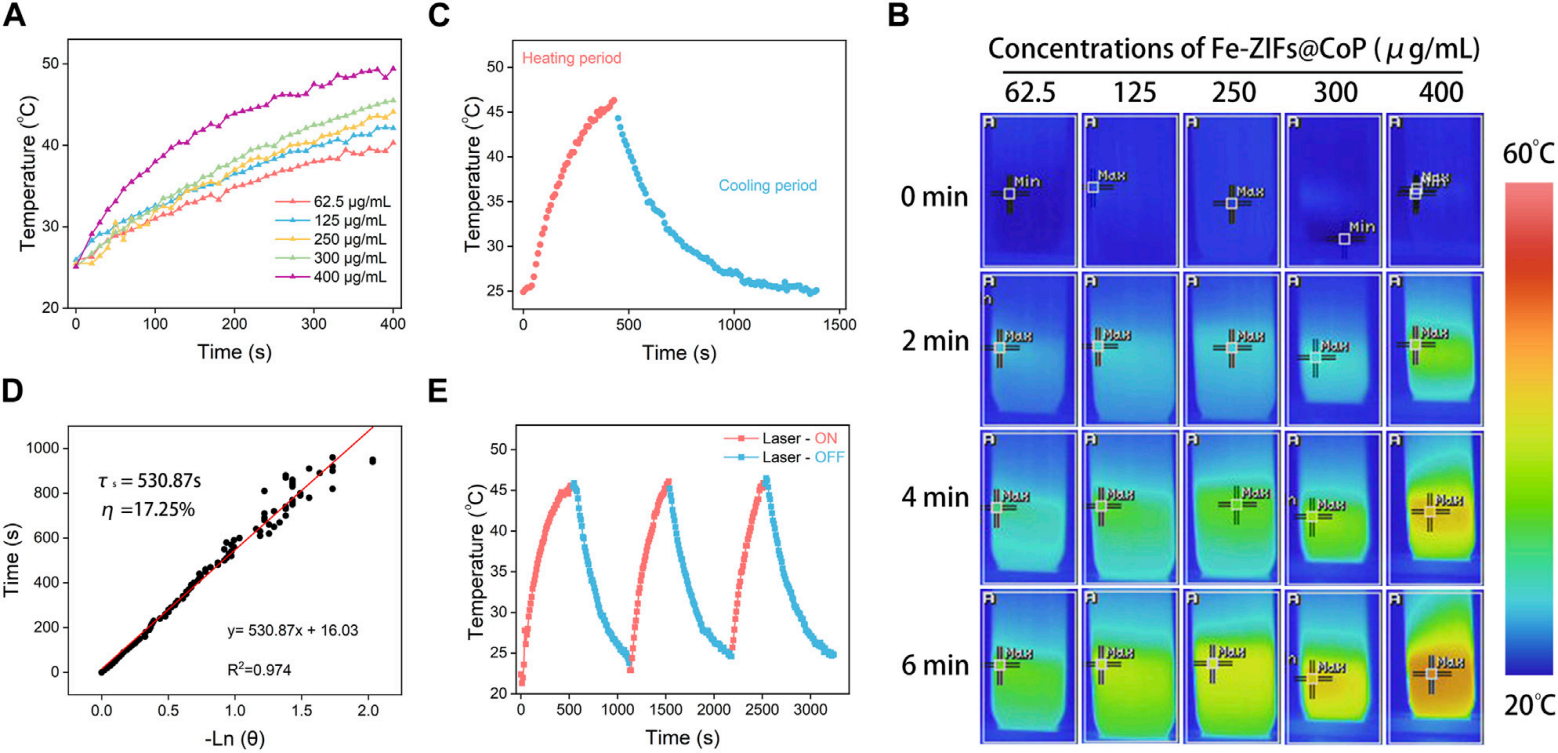
**(A)** The temperature increase of Fe-ZIFs@CoP under different concentrations. **(B)** Infrared thermal images of Fe-ZIFs@CoP under different concentrations. **(C)** The heating and cooling process. **(D)** Fitting linear relationship of -ln(𝜃)-t. **(E)** Recycling course.

The photothermal performance of Fe-ZIFs@CoP irradiated by an 808 nm laser is exhibited in Figure 3. The ultraviolet-visible (UV-vis) absorption spectrum of Fe-ZIFs@CoP was exhibited in Supplementary Figure S4. The results demonstrated the good absorption properties of Fe-ZIFs@CoP. To verify the photothermal performance, the photothermal conversion efficiency (*η*) of Fe-ZIFs@CoP was evaluated. In Figure 3A, the increased temperature of the Fe-ZIFs@CoP solution exhibited a dependence on the concentration manner. Typical infrared thermal images are presented in Figure 3B. The temperature of Fe-ZIFs@CoP solution at a concentration of 400 μg mL^−1^ can rise from 25.0°C to 49.5 °C in 400 s under 0.8 W cm^−2^ laser irradiation, indicating that the thermal energy was converted by Fe-ZIFs@CoP rapidly. Deriving from the cooling period in Figure 3C, the time constant (τ_s_) of Fe-ZIFs@CoP was determined to be 530.87 s, and the photothermal conversion efficiency under 808 nm irradiation is 17.25% (Figure 3D). The results demonstrate the excellent photothermal performance of Fe-ZIFs@CoP under NIR laser irradiation. Finally, the photothermal stability of Fe-ZIFs@CoP was assessed (Figure 3E). The Fe-ZIFs@CoP showed excellent photothermal stability with negligible attenuation of temperature after three heating/cooling cycles.

### 3.4 *In Vitro* anti-tumor properties

**FIGURE 4:**
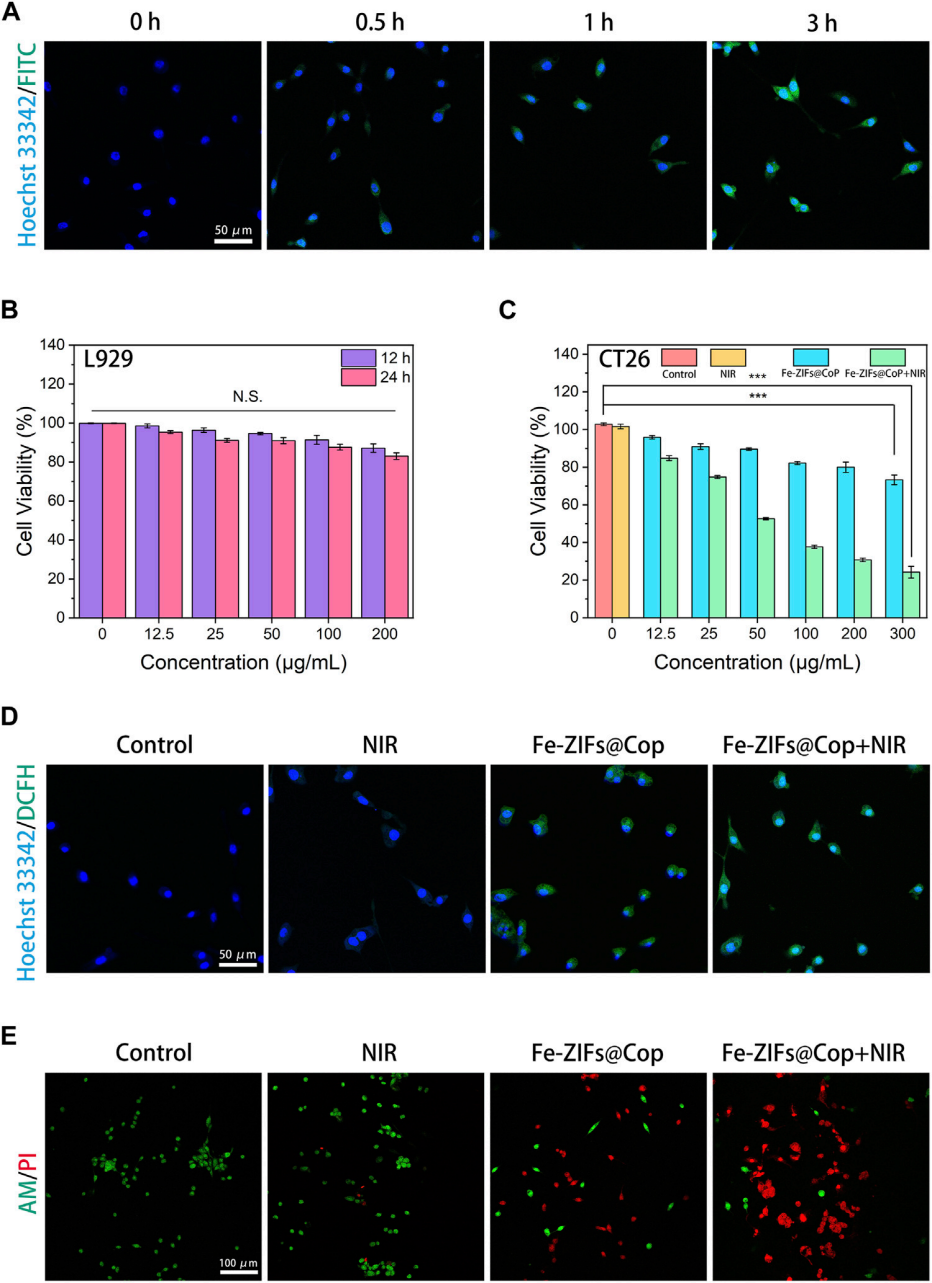
**(A)** CT26 cells uptake behavior. **(B, C)** Cell viabilities of Fe-ZIFs@CoP treated L929 cells and CT26 cells. *p < 0.05, **p < 0.01, ***p < 0.001. Data presented as mean ± S.D. (*n* = 5) **(D)** DCFH-DA staining of CT26 cells with different treatments. **(E)** Live/dead staining of CT26 cells.

The above characterization data confirmed the Fenton properties, GSH depletion, and photothermal effect of Fe-ZIFs@CoP. Inspired by these performances, the therapeutic effects of Fe-ZIFs@CoP at the cellular level were investigated. Before *in vitro* anti-tumor estimation, the cellular colocalization experiments of fluorescein isothiocyanate (FITC)-labeled Fe-ZIFs@CoP with the nucleus (labeled by Hoechst 33,342) were carried out to assess the ability of cells to phagocytose the material by a CLSM observation. As shown in Figure 4A, after incubation with CT26 cells for different times, the fluorescence of FITC-labeled Fe-ZIFs@CoP gradually appears and brightens, suggesting that the material could enter the tumor cells. Then, the cytotoxicity of Fe-ZIFs@CoP toward L929 fibroblast cells and CT26 colon cancer cells was recorded using the standard methylthiazolium tetrazolium (MTT) method. In Figure 4B, the survival rate of L929 cells co-incubation with different concentrations of Fe-ZIFs@CoP after 12 and 24 h was high, even with a high concentration of 200 μg/mL, demonstrating the good biosafety and biocompatibility of Fe-ZIFs@CoP. Then, the *in vitro* cytotoxicity was determined by CT26 colon cancer cells with varied treatments (Figure 4C). Compared with the control group, the NIR group showed negligible inhibition of cell viability, indicating the safety of the excitation source. However, the cells cultivated with Fe-ZIFs@CoP exhibited a noticeable death rate, originating from ROS generation and GSH depletion. It is worth noting that the viabilities of cells treated with Fe-ZIFs@CoP under 808 nm laser irradiation declined sharply. In addition, the activity of the cells decreased in a Fe-ZIFs@CoP concentration-dependent manner, thus, efficient cytotoxicity was achieved. The ROS-generating ability of Fe-ZIFs@CoP in CT26 cells was investigated by using DCFH-DA as a ROS probe. Thereafter, the fluorescent intensity of green fluorescent 2,7-dichlorofluorescein can indicate the amount of intracellular ROS. In Figure 4D, the CLSM images of cells treated with the control group or NIR group showed almost no green fluorescence owing to no production of ROS. Conversely, the cells treated with Fe-ZIFs@CoP and 808 nm laser emitted significantly enhanced fluorescence, even much stronger than that of cells treated with non-excited Fe-ZIFs@CoP, since limited ROS were generated in cells by the Fenton properties of Fe-ZIFs@CoP alone, while much more ROS were produced by both the photothermal effect-enhanced Fenton properties after the introduction of 808 nm laser. The ROS generation properties of Fe-ZIFs@CoP were also investigated by a pate reader, which the results were consistent with the above results (Supplementary Figure S5).

To reveal the overall effect of the above actions, calcein-AM and PI were used to co-stain CT26 cells. As we expected, in Figure 4E, the CLMS images showed that the cells were alive in the treatment groups of control and NIR nm, as evidenced by the strong green fluorescence. Conversely, the cells in the Fe-ZIFs@CoP group showed a decrease in green fluorescence and an increase in red fluorescence due to the therapeutic effect. More impressively, the cells in the Fe-ZIFs@CoP + NIR group showed a large amount of red fluorescence, largely benefiting from the effective therapies. The quantitative analysis of the live/dead staining results was also provided in Supplementary Figure S6 for ensuring the live/dead results.

### 3.5 *In Vivo* anti-tumor properties

**FIGURE 5:**
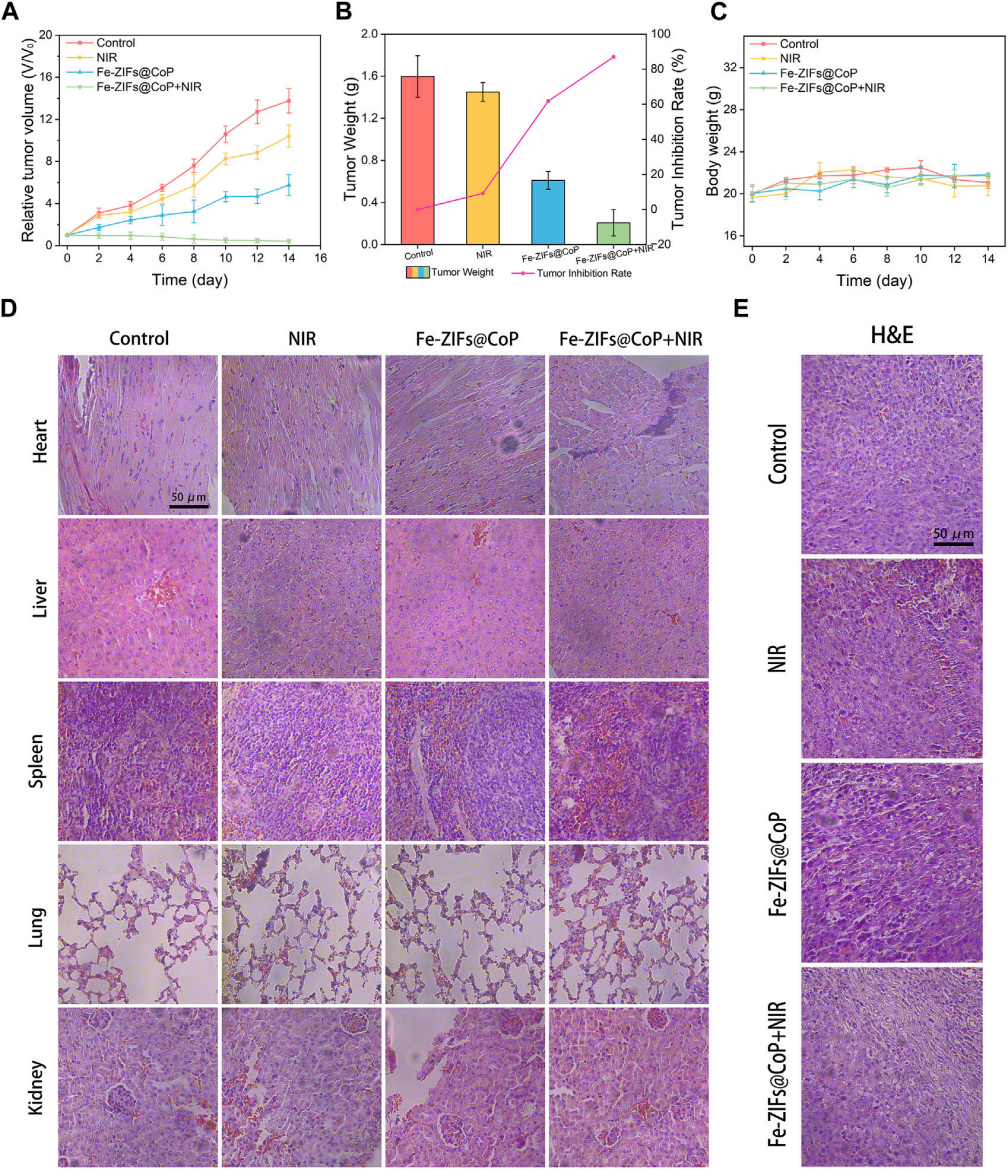
*In vivo* anti-tumor performance. **(A)** Relative tumor volume of different treatment groups. **(B)** Tumor weight of different treatment groups. **(C)** Body weight of the mice in different groups. **(D, E)** H&E staining images of major organs and tumors in different groups.

The *in vivo* anti-tumor effect of the Fe-ZIFs@CoP was further investigated. Firstly, the biodistribution of Fe-ZIFs@CoP within the body was examined. The major organs and tumors were collected for analysis and evaluation of their biodistribution by measuring Fe concentrations by ICP-OES (Supplementary Figure S7). Fe-ZIFs@CoP will be taken up by the spleen and liver, resulting in high concentrations of Fe in these organs. The concentration of Fe in the tumor increased with time and peaked at 12 h, suggesting that Fe-ZIFs@CoP could achieve good tumor targeting and accumulation. Then, the CT26 tumor-bearing mouse model was established. When the tumor volume reached 100 mm^3^, 200 µL of Fe-ZIFs@CoP dispersion was injected into mice *via* the tail vein. For evaluating the *in vivo* assessment of the therapeutic effect, twenty mice with a tumor size of 100 mm^3^ were divided into the following treatment groups: (1) control, (2) NIR, (3) Fe-ZIFs@CoP, and (4) Fe-ZIFs@CoP + NIR. Typically, mice in groups (3) and (4) were injected with 0.2 mL of Fe-ZIFs@CoP solution (2 mg kg^−1^) *via* the tail vein. Then, mice in groups (3) and (4) were irradiated by 808 nm (0.8 W cm^−2^) light at certain time points for therapy. The *in vivo* photothermal properties was exhibited in Supplementary Figure S8, which demonstrated the effective photothermal effect of Fe-ZIFs@CoP. The relative tumor volume of each mouse during the 14 days of treatment was recorded (Figure 5A), and the average tumor weight of each group was derived (Figure 5B). Significantly, the tumor growth rate of the control group was rapid, and the tumor volume of the NIR group did not show an effective growth inhibition. However, the mice in the Fe-ZIFs@CoP + NIR group showed an obvious tumor inhibition effect, which can be attributed to the effective therapeutic effect of the Fenton properties, photothermal effect, and photothermal-effect enhanced chemodynamic therapy. The representative tumor photograph of different group was exhibited in Supplementary Figure S9. Figure 5C presented the changes in body weight of the mice after treatment. There is no apparent weight fluctuation in all mice, which demonstrated that the treatments had no significant side effects on the survival status of the mice. After 14 days of treatment, major organs (Figure 5D) and tumors (Figure 5E) were dissected, photographed, and stained with H&E for histological examination. No morphological changes were observed in the tissue sections of other major organs, further demonstrating the safety of Fe-ZIFs@CoP. For the tumor tissue sections, no significant damage was observed in the control and NIR groups, yet local damage was observed in the Fe-ZIFs@CoP group, specifically a distinct cell death was showed in the Fe-ZIFs@CoP + NIR group. It can be concluded that Fe-ZIFs@CoP activated by 808 nm radiation achieve effective tumor suppression through synergistic Fenton properties, GSH depletion, and photothermal effect.

## 4 Conclusion

In summary, we present the Fe-ZIFs@CoP nanoplatforms by taking advantage of MOFs, which were applied for Fenton properties, photothermal effect, and photothermal-effect enhanced chemodynamic therapy. Under 808 nm laser, large amounts of ROS could be generated due to the photothermal enhanced Fenton properties of Fe-ZIFs@CoP. While glutathione could also be depleted to amplify oxidative stress. Our study may provide some prospects for MOFs-based nanoplatforms for future directions in tumor therapy.

## Data Availability

The original contributions presented in the study are included in the article/Supplementary Material, further inquiries can be directed to the corresponding author.
