# Supplementary material for: Rational designed Fe-ZIFs@CoP nanoplatforms for photothermal-enhanced ROS-mediated tumor therapy
Source: Front Bioeng Biotechnol. 2024 Jan 31;12:1361347. doi: 10.3389/fbioe.2024.1361347 (PMC10865240; doi:10.3389/fbioe.2024.1361347)
Supplement: Supplementary file 1 [file DataSheet1.docx]

**Supporting Information**

**Rational designed Fe-ZIFs@CoP nanoplatforms for photothermal-enhanced ROS-mediated tumor therapy**

*Chen Wang^1,2^; Shufang Ning^2^; Jinling Mai^3^; Shanyu Zhao^2^; Wenwei Jiang^2^; Junjie Pan^2^; Feifei Wu^2^; Qiuju Liu^2^; Qinle Zhang^1*^*

^1^ Maternal and Child Health Hospital of Guangxi Zhuang Autonomous Region, Nanning 530021, China

^2^ Guangxi Medical University Cancer Hospital, Nanning 530021, China

^3^ The First Affiliated Hospital of Guangxi Medical University, Nanning 530021, China

*** Correspondence:**

Qinle Zhang
E-mail: [qinlezhang@hotmail.com](mailto:qinlezhang@hotmail.com)


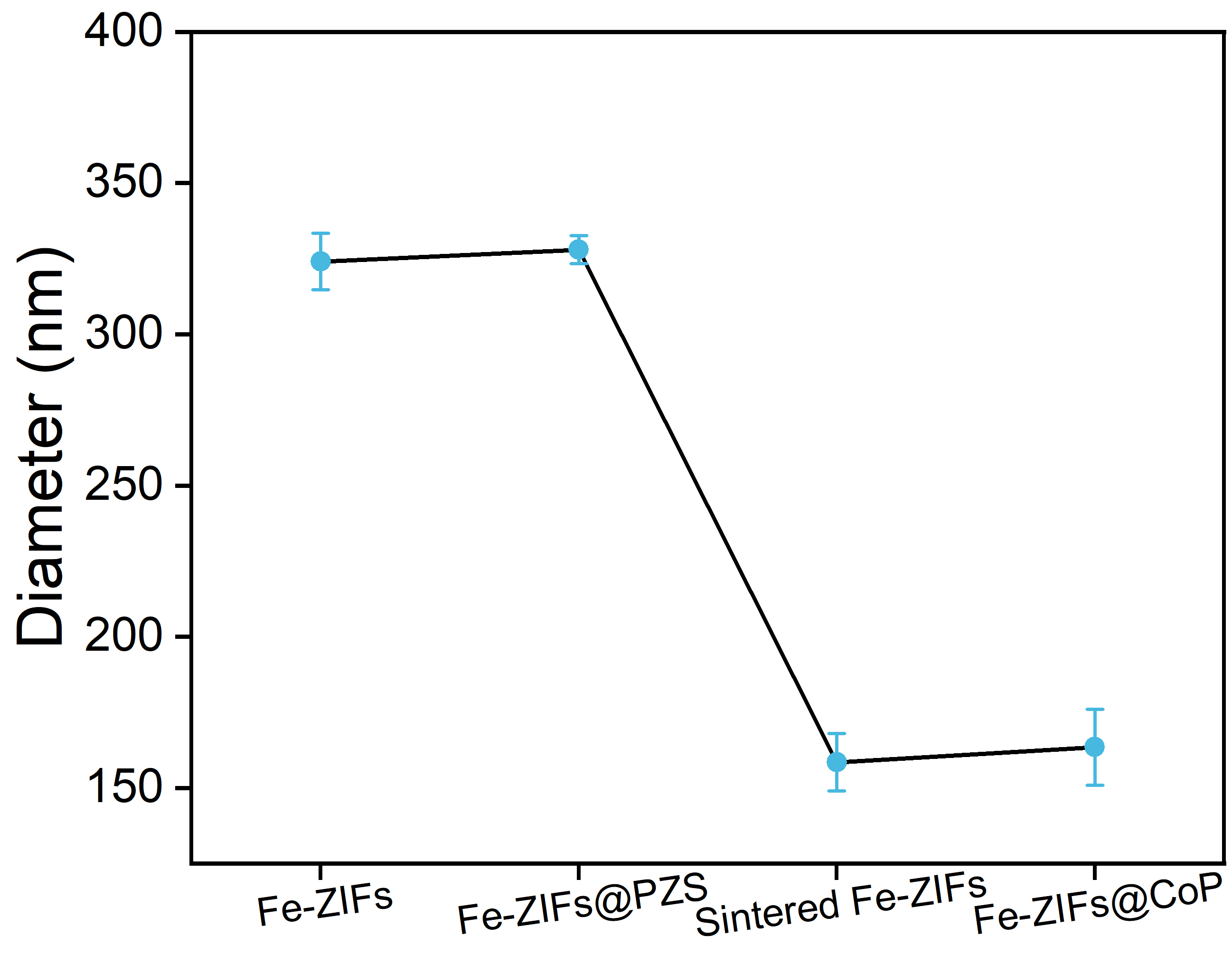


Figure S1 The size distribution of Fe-ZIFs@CoP.


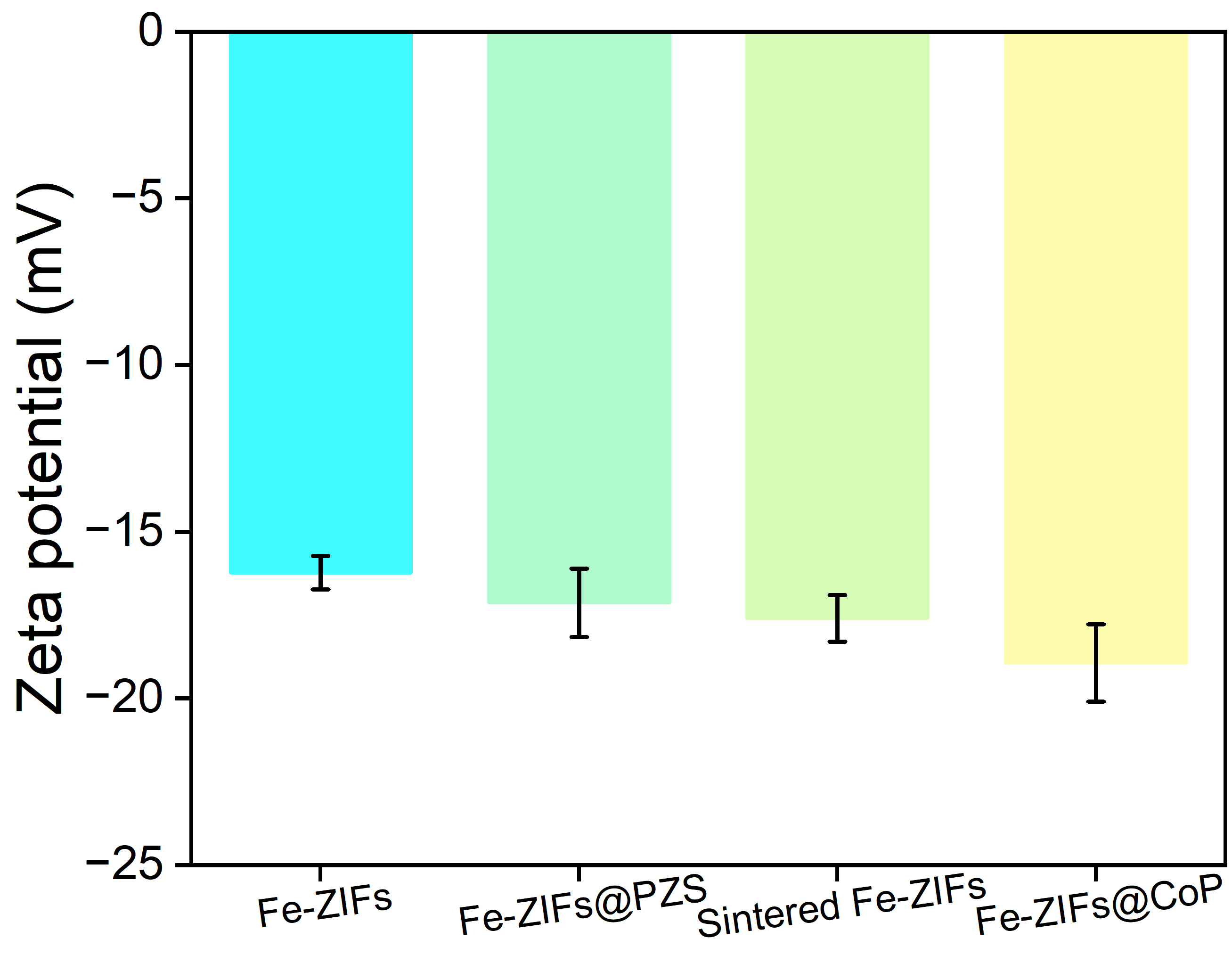


Figure S2 The zeta potential of Fe-ZIFs, Fe-ZIFs@PZS, sintered Fe-ZIFs, and Fe-ZIFs@CoP.


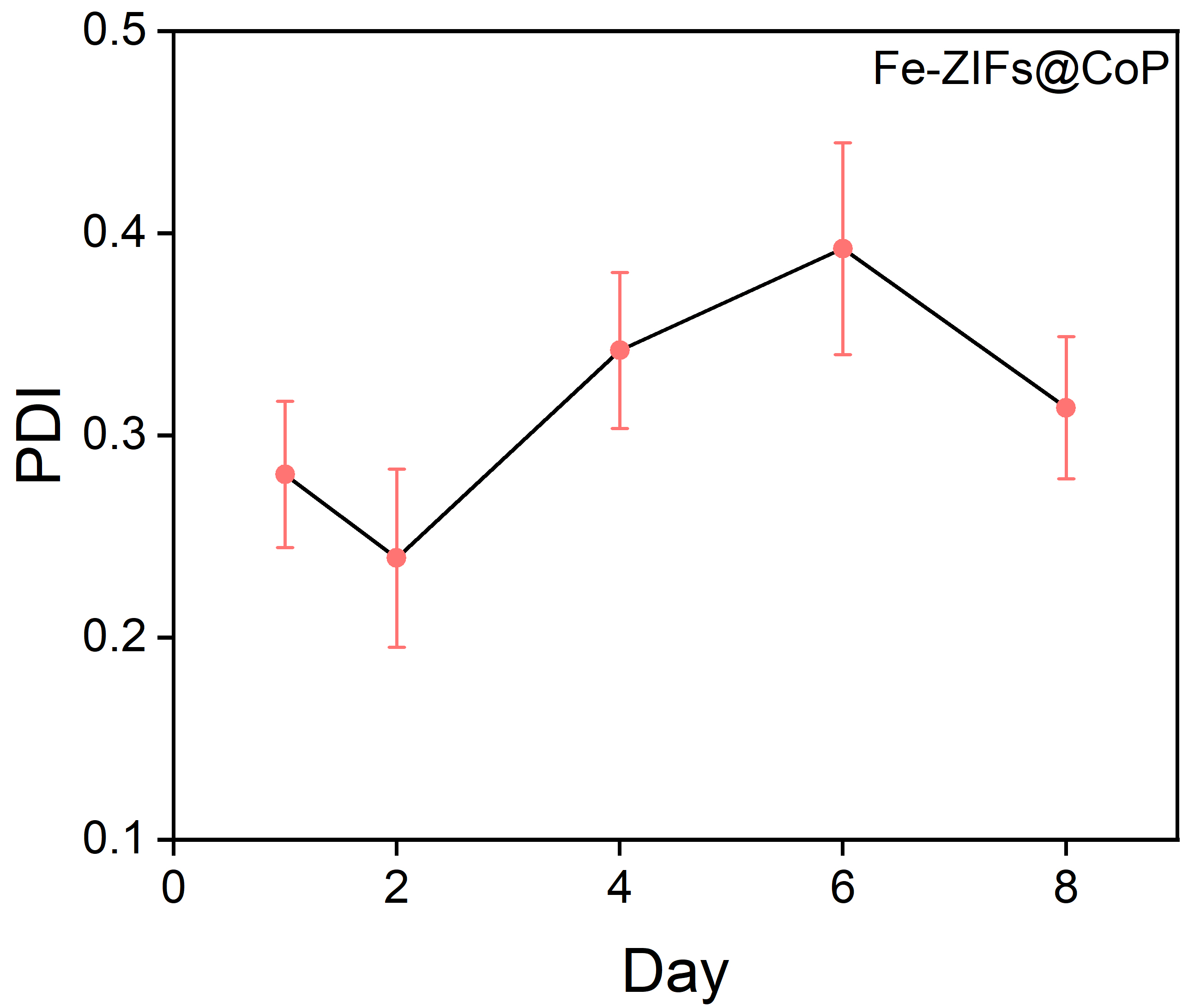


Figure S3 The stability properties of Fe-ZIFs@CoP.


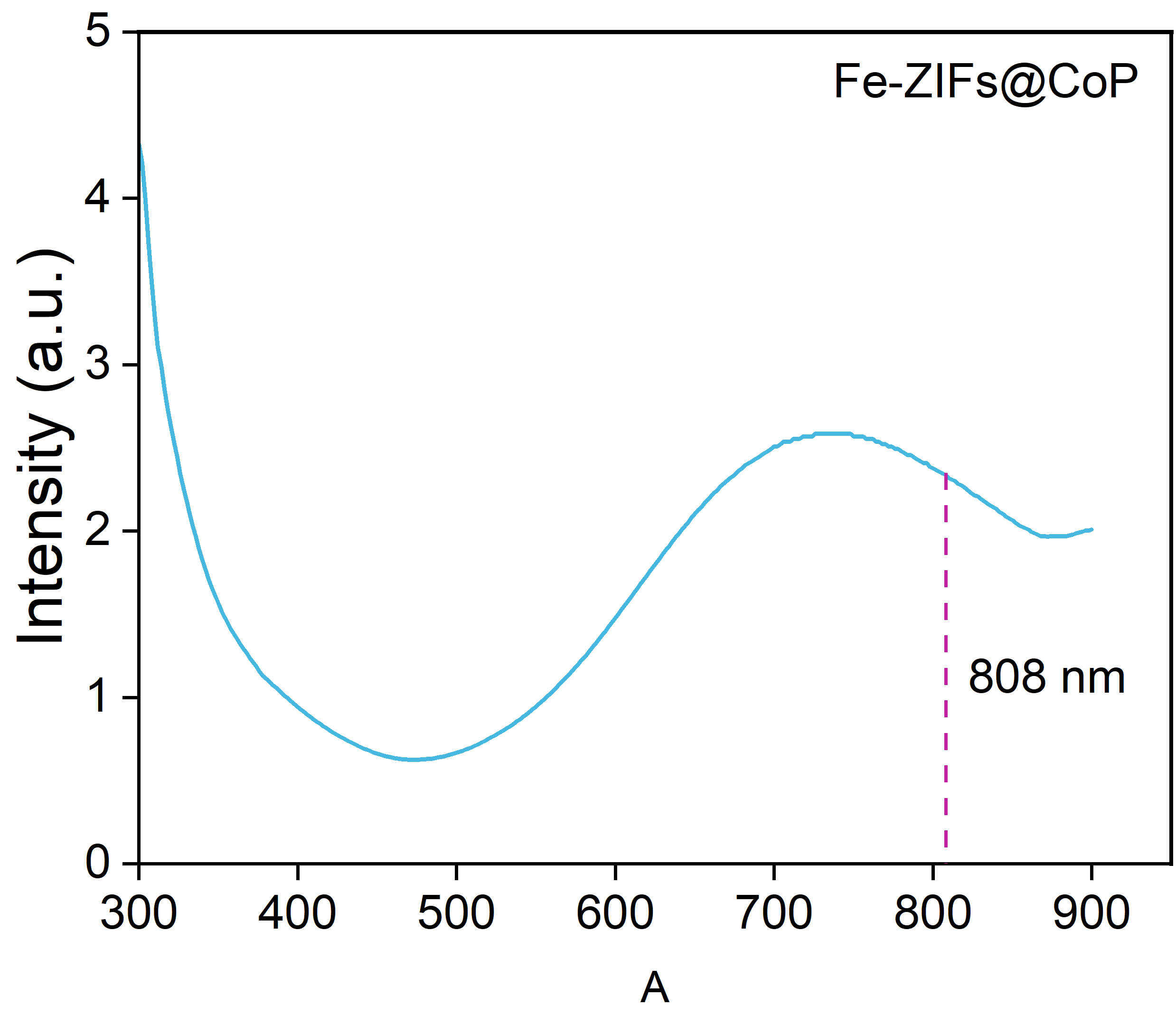


Figure S4 The UV-vis absorption spectrum of Fe-ZIFs@CoP.


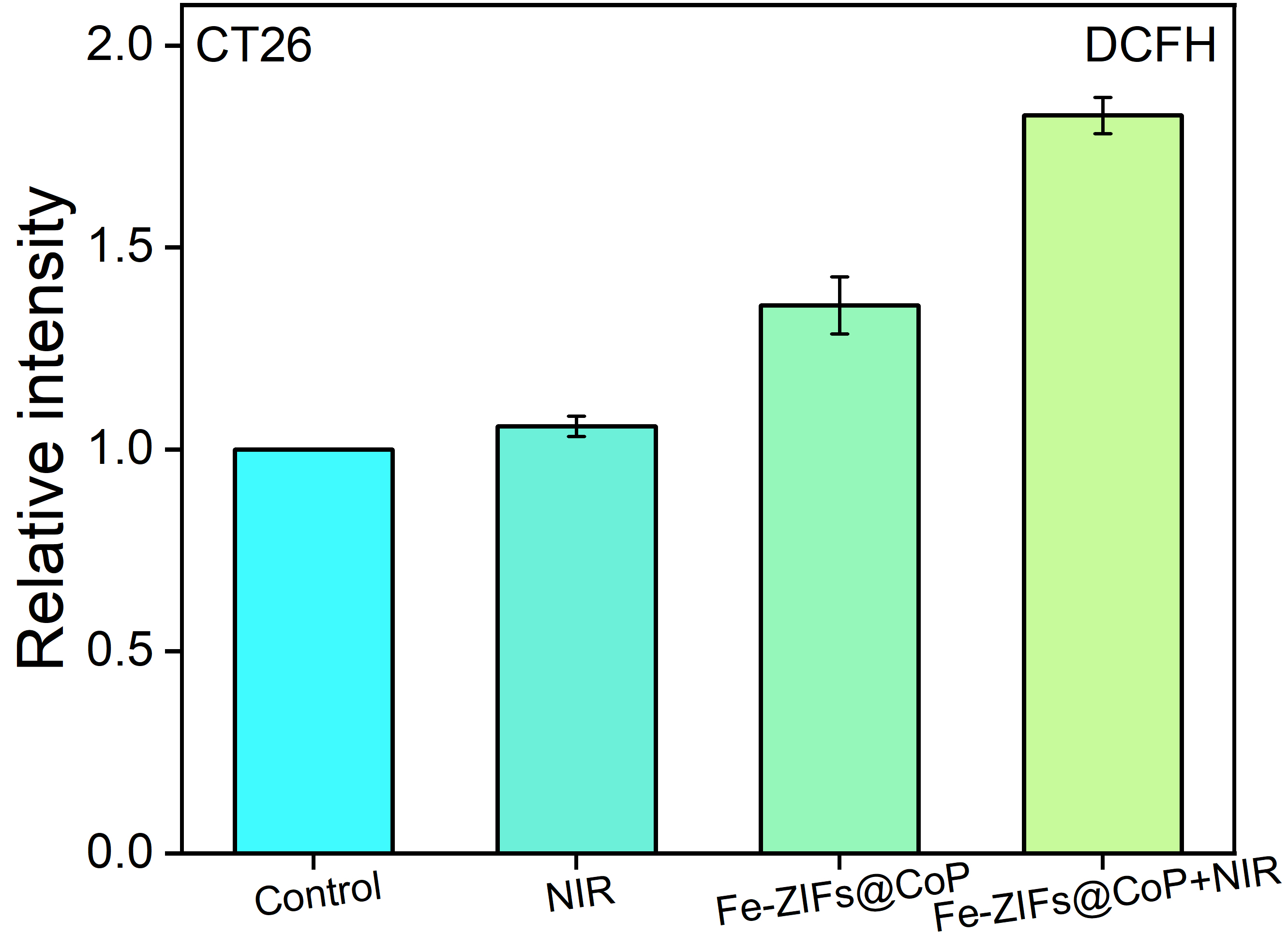


Figure S5 The ROS generation properties of Fe-ZIFs@CoP.


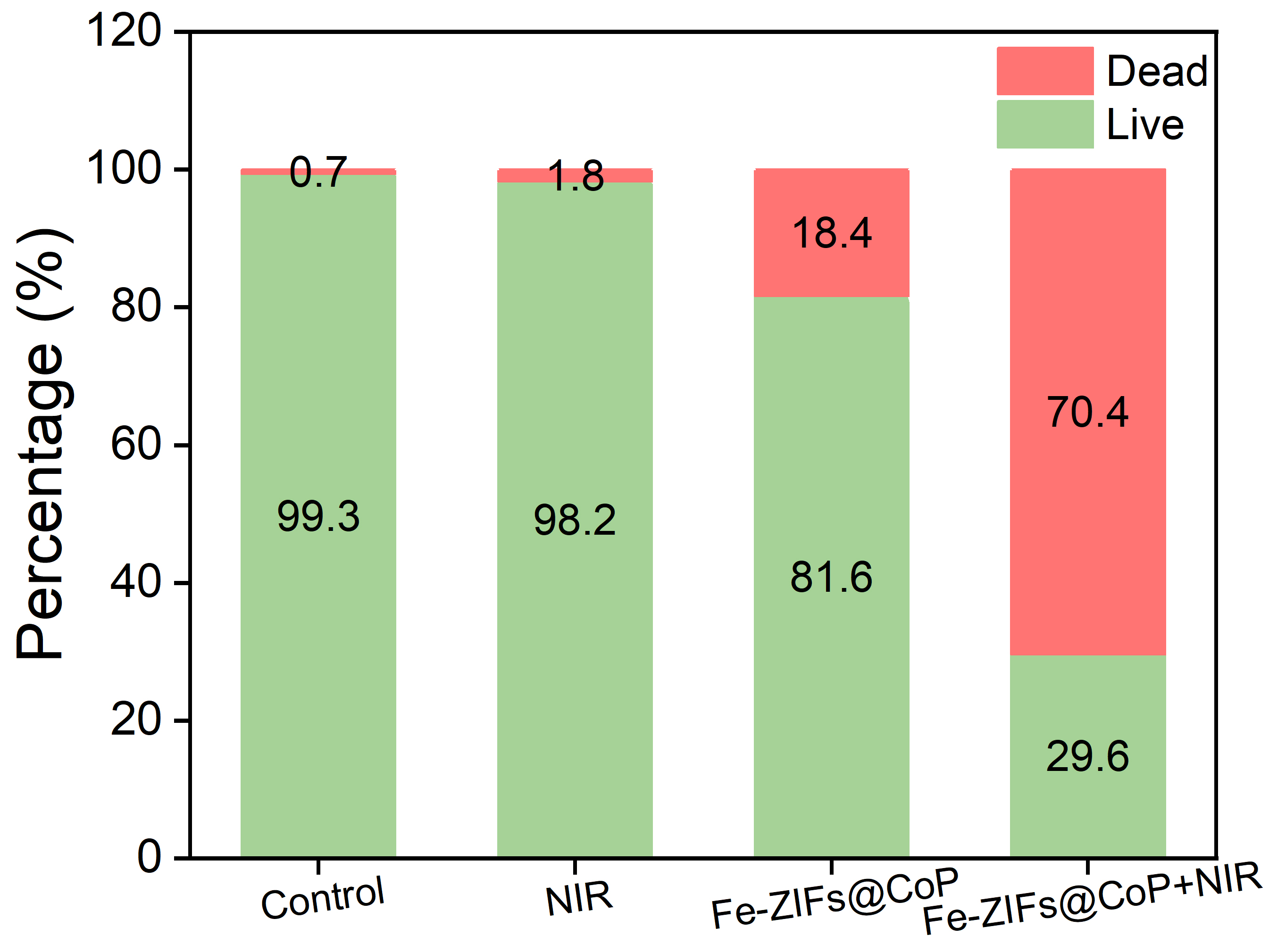


Figure S6 The quantitative analysis of the live/dead staining results.


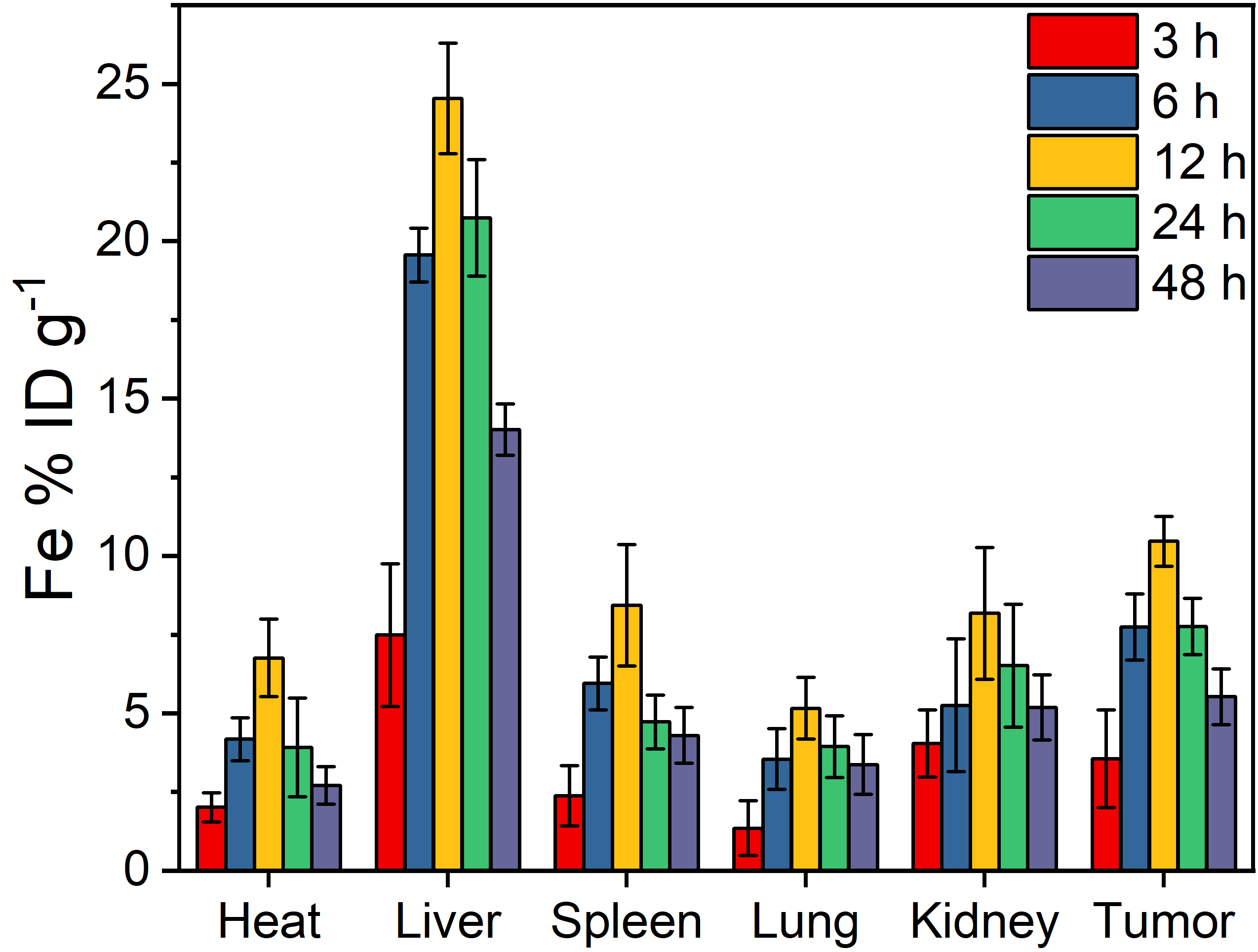


Figure S7 The metabolism properties of Fe-ZIFs@CoP *in vivo*.


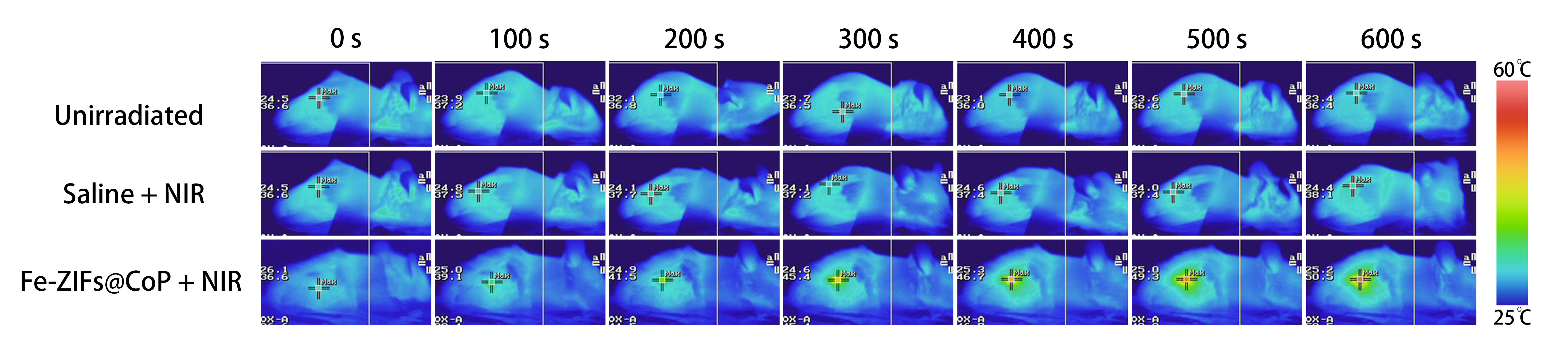


Figure S8 The photothermal properties of Fe-ZIFs@CoP *in vivo*.


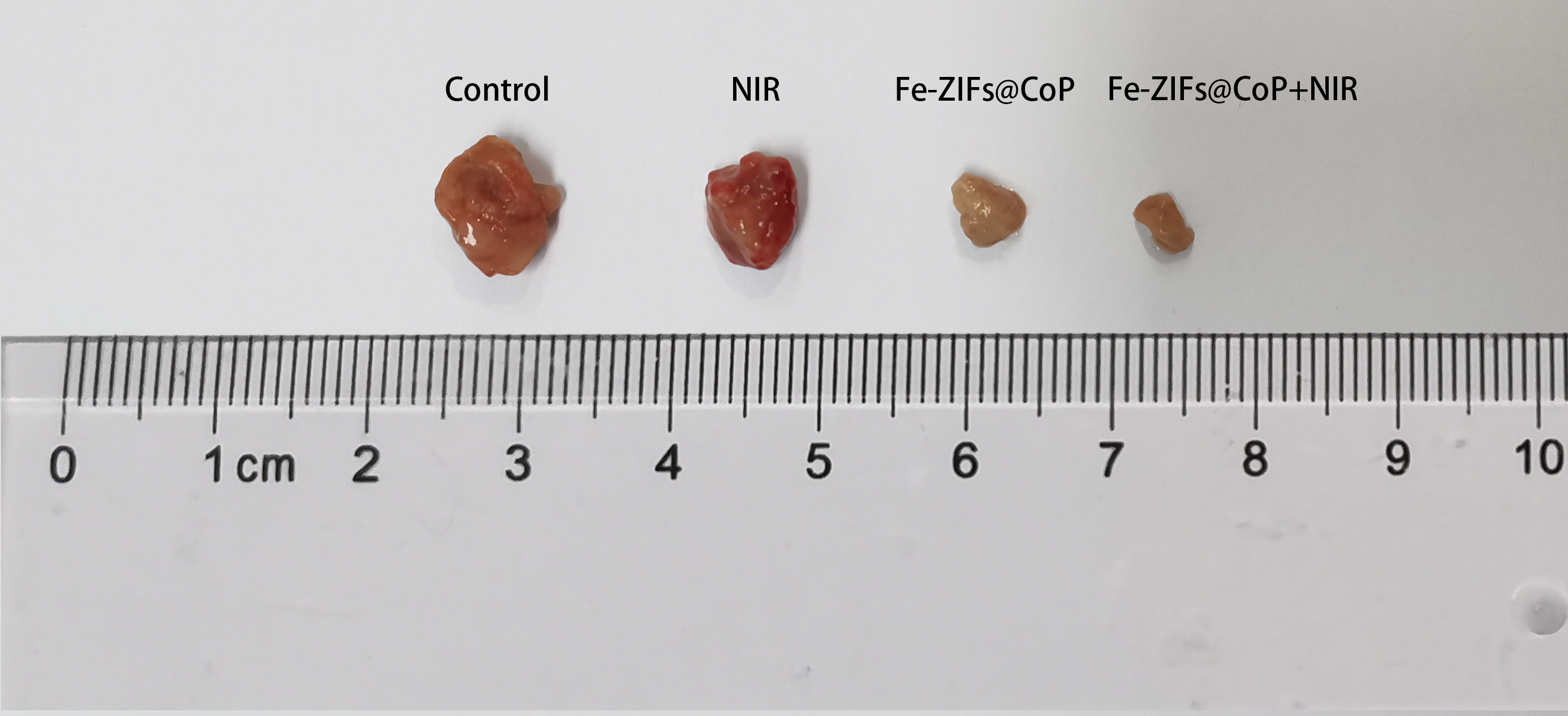


Figure S9 The representative photograph of tumors in different groups after treatment.
